# Cardiovascular magnetic resonance pulmonary perfusion for guidance of interventional treatment of pulmonary vein stenosis

**DOI:** 10.1186/s12968-022-00904-x

**Published:** 2022-12-12

**Authors:** Cosima Jahnke, Andreas Bollmann, Sabrina Oebel, Frank Lindemann, Ingo Daehnert, Frank-Thomas Riede, Gerhard Hindricks, Ingo Paetsch

**Affiliations:** 1 Department of Electrophysiology, Heart Center Leipzig at University of Leipzig, Struempellstr. 39, 04289 Leipzig, Germany; 2 Department of Pediatric Cardiology, Heart Center Leipzig at University of Leipzig, Leipzig, Germany

**Keywords:** Cardiovascular magnetic resonance imaging, Pulmonary vein stenosis, Pulmonary perfusion, Pulmonary vein stenting, Pulmonary vein isolation

## Abstract

**Background:**

Pulmonary vein (PV) stenosis represents a rare but serious complication following radiofrequency ablation of atrial fibrillation with a comprehensive diagnosis including morphological stenosis grading together with the assessment of its functional consequences being imperative within the relatively narrow window for therapeutic intervention. The present study determined the clinical utility of a combined, single-session cardiovascular magnetic resonance (CMR) imaging protocol integrating pulmonary perfusion and PV angiographic assessment for pre-procedural planning and follow-up of patients referred for interventional PV stenosis treatment.

**Methods:**

CMR examinations (cine imaging, dynamic pulmonary perfusion, three-dimensional PV angiography) were performed in 32 consecutive patients prior to interventional treatment of PV stenosis and at 1-day and 3-months follow-up. Degree of PV stenosis was visually determined on CMR angiography; visual and quantitative analysis of pulmonary perfusion imaging was done for all five lung lobes.

**Results:**

Interventional treatment of PV stenosis achieved an acute procedural success rate of 90%. Agreement between visually evaluated pulmonary perfusion imaging and the presence or absence of a ≥ 70% PV stenosis was nearly perfect (Cohen’s kappa, 0.96). ROC analysis demonstrated high discriminatory power of quantitative pulmonary perfusion measurements for the detection of ≥ 70% PV stenosis (AUC for time-to-peak enhancement, 0.96; wash-in rate, 0.93; maximum enhancement, 0.90). Quantitative pulmonary perfusion analysis proved a very large treatment effect attributable to successful PV revascularization already after 1 day.

**Conclusion:**

Integration of CMR pulmonary perfusion imaging into the clinical work-up of patients with PV stenosis allowed for efficient peri-procedural stratification and follow-up evaluation of revascularization success.

**Supplementary Information:**

The online version contains supplementary material available at 10.1186/s12968-022-00904-x.

## Background

Pulmonary vein (PV) stenosis represents a rare but severe complication following radiofrequency ablation of atrial fibrillation [1, 2]. Delayed and incorrect diagnosis occurs frequently due to a non-specific clinical presentation mimicking other more common pulmonary and cardiac illnesses [3]. Thus, an expedient and comprehensive diagnosis is imperative within the relatively narrow window for therapeutic intervention. Beside the mere angiographic evidence of severe PV stenosis, the hemodynamic consequences on the levels of parenchymal lung perfusion and right-ventricular (RV) function need to be taken into account, in order to achieve an objective and profound clinical decision making. Cardiovascular magnetic resonance (CMR) imaging is ideally suited to provide all desired morphological and functional information within a single-session examination with the additional advantage of radiation-free follow-up examinations. Consequently, the present study evaluated the ability of a comprehensive CMR protocol integrating RV function assessment, pulmonary perfusion imaging and three-dimensional PV angiography for stratification of PV stenosis patients referred for interventional treatment and assessment of interventional success during a 3-months follow-up.

## Methods

### Patient population

Consecutive patients scheduled for interventional treatment of a symptomatic PV stenosis following radiofrequency ablation were included; presence of significant PV stenosis was assessed by either CMR angiography, computed tomography (CT) angiography or X-ray angiography, respectively. CMR imaging was performed prior to PV intervention and repeated 1 day and 3 months post successful interventional treatment. Clinical evaluation was performed pre-intervention and 3 months post-intervention. The study was conducted in accordance with the local institutional review board and the standards of the University of Leipzig ethics committee; written informed consent was obtained from all patients.

### CMR imaging protocol

A 1.5 T CMR scanner (Ingenia, Philips Healthcare, Best, The Netherlands) equipped with a 28-element array coil with full in-coil signal digitalization and optical transmission was used for all CMR examinations. The combined single-session CMR protocol consisted of cine imaging, pulmonary arterial flow measurement, pulmonary perfusion imaging and three-dimensional PV angiography. Cine image acquisition followed current recommendations (balanced steady-state free precession sequence; measured in-plane spatial resolution, 1.5 × 1.5 mm²; slice thickness, 8 mm; temporal resolution, 30–40 phases per cardiac cycle) and covered all standard cardiac geometries (i.e. multiple short axis views and a 4-, 3-, and 2-chamber view) [4]. Two-dimensional phase-contrast flow measurement was performed in the main pulmonary artery with the imaging plane 10 mm above the pulmonary valve and perpendicular to the flow direction (in-plane spatial resolution, 1.4 × 1.4 mm²; slice thickness, 10 mm; temporal resolution, 35 phases per cardiac cycle); velocity encoding was routinely set to 150 cm/s and adapted individually if needed.

High-resolution, contrast-enhanced dynamic pulmonary perfusion imaging was done in coronal slice orientation using *k-t* SENSE in combination with a saturation recovery gradient echo pulse sequence (repetition time/echo time, 2.5 ms/0.9 ms; flip angle, 15°; saturation prepulse delay, 110 ms; measured in-plane spatial resolution, 1.4 × 1.4 mm²; slice thickness, 12 mm; *k-t* factor of 6 with 11 *k-t* interleaved training profiles; 3 slices acquired per heartbeat; number of dynamics ranged from 12 to 24) [5]. Pulmonary perfusion imaging was carried out in end-expiration breath-holding using a peripheral intravenous injection of a gadoteric acid bolus (Dotarem^®^, Guerbet, Villepinte, France; 0.05 mmol/kg bodyweight; injection rate, 4.0 ml/s). Directly thereafter, contrast-enhanced three-dimensional CMR PV angiography was conducted during inspiratory breath-holding without electrocardiographic gating (coronal slice orientation; full coverage of the left atrium and the PVs; isotropic spatial resolution, 1.0 × 1.0 × 1.0 mm^3^; acquisition of 2 consecutive contrast-enhanced dynamics). Real‐time bolus tracking was applied for accurate timing during contrast agent bolus injection (gadoteric acid; 0.1 mmol/kg bodyweight; injection rate, 4.0 ml/s) [6].

### CMR image analysis

#### Cine imaging

Left ventricular (LV) and RV end-diastolic and end-systolic volumes (LVEDV; LVESV; RVEDV; RVESV) were assessed according to standard definitions and LV ejection fraction (LVEF) and RV ejection fraction (RVEF) was calculated [7]. The presence or absence of end-systolic septal flattening (D-shape pattern) indicating RV pressure overload was visually determined [8].

### Pulmonary artery flow measurement

Pulmonary artery contours were semi-automatically drawn and time-resolved pulmonary artery flow curves were displayed for visual evaluation. The presence or absence of a systolic notch being indicative of increased pulmonary vascular resistance was established.

### Dynamic pulmonary perfusion imaging

Pulmonary CMR perfusion imaging was evaluated by a certified CMR imaging expert (CJ, IP) being blinded to the results of CMR angiography and PV intervention. Pulmonary perfusion was visually evaluated for the presence or absence of relative hypoenhancement per pulmonary lobe. In addition, pulmonary perfusion was quantitatively analysed using a dedicated software analysis tool (IntelliSpace Portal 11.0, Philips Healthcare); a region of interest (ROI, > 300 mm²) was placed in each of the five lung lobes including peripheral pulmonary parenchyma only while carefully avoiding any cross-sections of segmental pulmonary arteries or veins. The following quantitative pulmonary perfusion parameters were derived from the signal-intensity time curves in each of the 5 lung lobes: maximum enhancement [arbitrary unit], time-to-peak enhancement [s], wash-in rate [s^−1^], and area-under-the-curve (AUC, [arbitrary unit]) with maximum enhancement representing the change in signal intensity from baseline, time-to-peak enhancement representing the time from contrast arrival to the maximum of the signal-intensity curve, and wash-in rate representing the tangent slope of the signal-intensity time curve. The average gain attributable to successful PV revascularization was determined by the differences of quantitative pulmonary perfusion measurements within the respective lung lobes between pre- and post-interventional assessments.

#### Three-dimensional pulmonary vein angiography

Pre-interventional imaging identified PV anatomy and the presence of PV side branches. Each PV ostium was visually evaluated with regard to the degree of stenosis (range, 0 to 100%). In patients with total or subtotal PV occlusion, additional evaluation of the second contrast-enhanced angiography dynamic acquisition determined the presence or absence of “late-filling” PV side branches. Only (sub)total PV occlusions with visible side-branches on the first dynamic and/or “late-filling” of peripheral side-branches on the second dynamic angiography scan were considered for subsequent revascularization. Volume-rendering reconstruction was performed and a surface mesh model was generated with the exported mesh data used for anatomical guidance during subsequent PV intervention.

During CMR follow-up examinations, implanted PV stents rendered direct angiographic evaluation of the corresponding ostial segments impossible due to stent-related artifacts. Thus, only visible PV side branches distal of the stent artifact were documented and angiographic grading was restricted to the non-stented PV ostia only.

### Invasive PV angiography and revascularization

Revascularization treatment was indicated in symptomatic patients with a PV stenosis ≥ 70% [9, 10]. PV interventions were performed under deep propofol sedation; after transseptal puncture, a steerable sheath was introduced into the left atrium to facilitate accurate delineation and intervention of the targeted PV ostium. Retrograde contrast-enhanced PV angiography allowed for confirmation and detailed assessment of PV stenosis. Interventional procedures were assisted by three-dimensional electro-anatomical mapping systems and reconstructed left atrial anatomy from CMR angiography. In general, the targeted PV stenoses were primarily dilated by careful balloon inflation followed by appropriately sized stent implantation to reduce the general risk of early re-stenosis [3, 10], Procedural success on a per patient level was defined by at least one successfully revascularized pulmonary vein.

### Statistical analysis

All analyses were done using SPSS (version 21, Statistical Package for the Social Sciences, International Business Machines, Inc., Armonk, New York, USA). Continuous variables were given as mean ± standard deviation for normally distributed data; frequencies and percentages were used to describe categorical data. Differences between continuous and categorical variables were assessed using Student’s t-test, related-samples Friedman’s two-way analysis of variance by ranks, Chi-square, or Cochran’s Q test as appropriate. All tests were two-tailed and a p-value of < 0.05 was considered significant.

Cohen’s kappa was applied to measure agreement between visually assessed pulmonary perfusion and the presence/absence of severe PV stenosis using the following grading: 0–0.2 (poor), 0.21–0.4 (fair), 0.41–0.6 (moderate), 0.61–0.8 (substantial), and 0.81–1.0 (nearly perfect) [11]. To determine the relationship between quantitative pulmonary perfusion analysis and the presence of a PV stenosis ≥ 70%, receiver-operator characteristic curve (ROC) analysis was performed and the area under the curve was calculated.

The treatment effect of successful PV revascularization on quantitative pulmonary perfusion was determined with Cohen’s d (effect size calculated as the difference of the means divided by the standard deviation) using the following scale: d = 0.2 indicates small; d = 0.5, medium; d = 0.8, large; d = 1.2, very large; d > 2.0, huge treatment effect.

## Results

### Patient population

Thirty-two study participants (27 male; 57 ± 12 years; body mass index (BMI), 29.1 ± 5.5 kg/m²) were enrolled with a total of 58 high-grade PV stenoses as defined by CMR angiography (i.e. PV stenosis ≥ 70%). Patients generally presented with reduced exercise capacity, progressive dyspnea (New York Heart Association (NYHA) II: 8/32, 25%; NYHA III: 24/32, 75%) or hemoptysis (7/32, 22%). Time from last PV ablation procedure to diagnosis of PV stenosis was 14.2 ± 13.1 months (range, 1–54 months) mainly following multiple PV ablation procedures (mean 2.2 ± 1.1; range, 1 to 5). Diagnosis of PV stenosis was established by either CMR angiography (16/32, 50%), prior CT angiography (11/32, 34%) or X-ray angiography during a redo catheter ablation procedure (5/32, 16%), respectively. Indication for the first ablation procedure was paroxysmal (11/32, 34%) or persistent atrial fibrillation (21/32, 66%). During pre-interventional CMR imaging, patients presented mainly with sinus rhythm (26/32, 81%) or atrial fibrillation (4/32, 13%) and atypical atrial flutter (2/32, 6%), respectively.

**Fig. 1:**
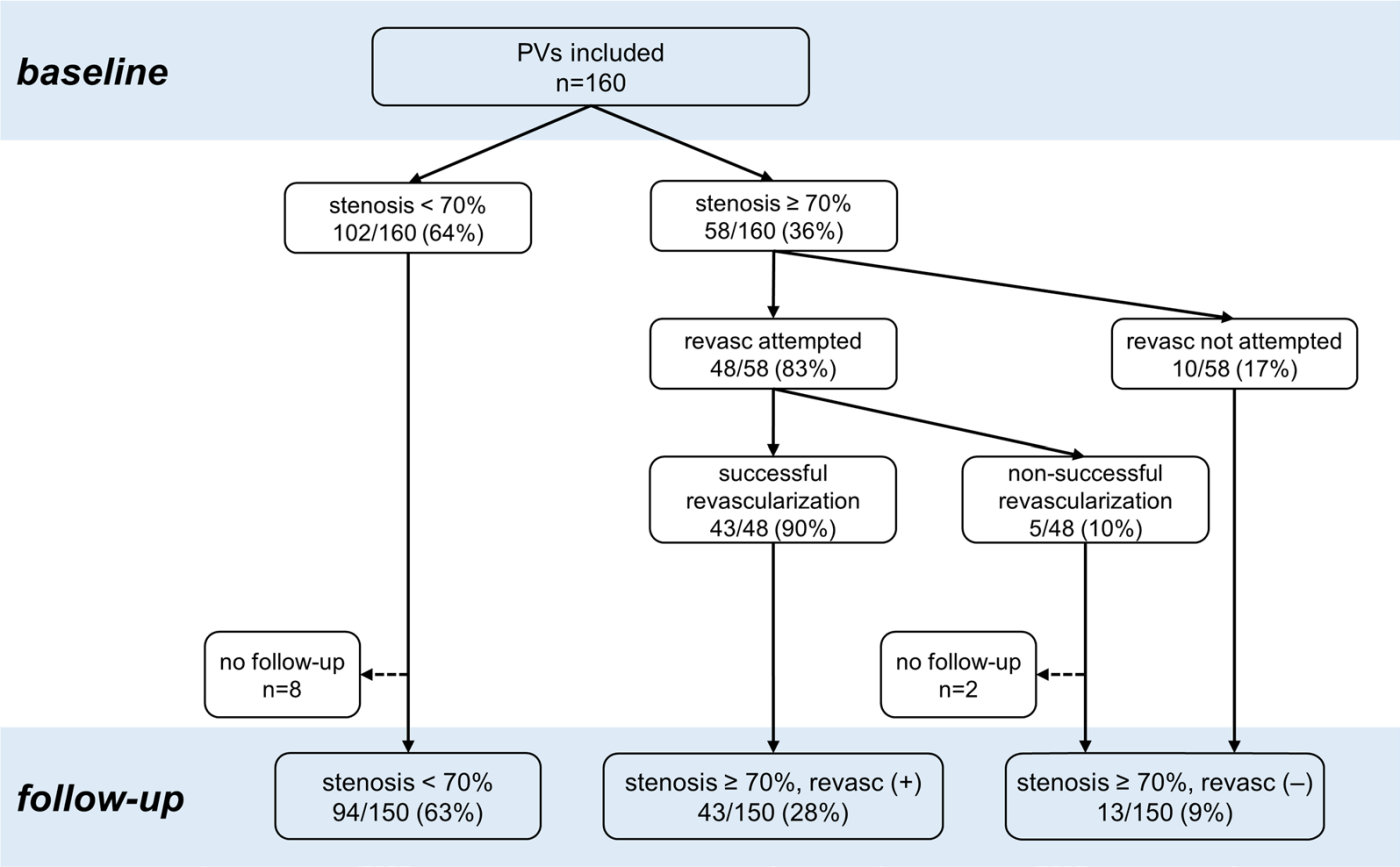
Peri-procedural stratification of the study cohort. Flow chart illustrating the peri-procedural stratification of the study population forming three subgroups considered during the post-interventional follow-up period. Two patients with a non-successful revascularization attempt of a single pulmonary vein (PV) occlusion did not undergo post-interventional follow-up cardiovascular magnetic resonance (CMR) examinations corresponding to 2 PVs “stenosis ≥ 70%, revasc (‒)” and 8 PVs “stenosis < 70%”. PV indicates pulmonary vein; revasc, revascularization

In two patients, each with a long segment occlusion of one PV (occlusion length, 16 and 20 mm, respectively), revascularization was attempted but did not succeed and, thus, both patients were not invited for post-interventional follow-up CMR examinations. Three patients underwent only a clinical follow-up without CMR imaging after 3 months: one patient with a pacemaker and two patients with a distant residence, who made follow-up appointments with their primary care physician. Figure 1 illustrates the composition of the study cohort.

### Baseline CMR imaging

Detailed results of cine and pulmonary flow imaging are given in Table 1. CMR angiography revealed a severe stenosis of a single PV in 14 patients (44%), while in all other patients multiple PVs were affected: ≥ 70% stenoses of two PVs in 12 patients (37%), of three PVs in five patients (16%), and of all five PVs in one patient (3%). Anatomical distribution of high-grade stenosis was significantly different with the left superior PV (LSPV) being the most commonly affected PV (p = 0.005): LSPV stenosis was seen in 56% (18/32) of patients, left inferior PV (LIPV) stenosis in 47% (15/32), right superior PV (RSPV) stenosis in 38% (12/32), right middle PV (RMPV) stenosis in 28% (9/32), and right inferior PV (RIPV) stenosis in 13% (4/32). In general, left-sided PVs were more frequently involved than right-sided PVs (75%, 24/32 vs. 50%, 16/32; p = 0.001) and superior PVs more frequently than inferior PVs (81%, 26/32 vs. 53%, 17/32; p = 0.011).

**Table 1:** CMR imaging parameters

	Preintervention	1-day postintervention (n = 30)	3-months postintervention (n = 27)	p-value
Heart rate, bpm	72 ± 14	68 ± 13	63 ± 9	0.005
Systolic blood pressure, mmHg	133 ± 14	130 ± 14	133 ± 15	0.756
LVEDV, ml	143 ± 34	147 ± 29	153 ± 30	0.698
LVEF, %	60 ± 5	59 ± 5	60 ± 4	0.555
RVEDV, ml	151 ± 49	151 ± 45	153 ± 45	0.990
RVEF, %	59 ± 10	60 ± 9	59 ± 9	0.043
D-shape	9 (28)	4 (13)	3 (11)	0.015
Systolic notch	13 (41)	7 (23)	7 (26)	0.030
LA, cm²	22 ± 6	22 ± 5	23 ± 3	0.418
RA, cm²	22 ± 4	23 ± 4	23 ± 3	0.542

Values are mean ± SD or n (%); p-values are given for the comparison of baseline vs. follow-up data

*LA* left atrium, *LV* left-ventricular, *LVEDV* left ventricular end-diastolic volume, *LVEF* left ventricular ejection fraction, *RA* right atrium, *RV* right-ventricular, *RVEDV* right ventricular end-diastolic volume, *RVEF* right ventricular ejection fraction

**Fig. 2:**
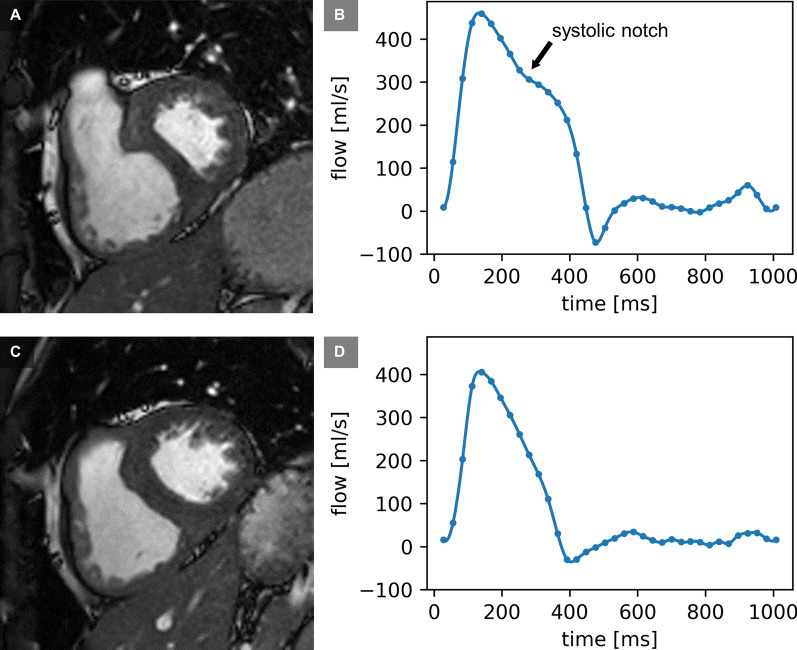
Cine CMR and pulmonary artery flow imaging for depiction of left ventricular (LV) “D-shape” pattern and systolic notching. **A**, **B** Systolic still frame of cine short axis view at baseline (**A**). Systolic flattening of the interventricular septum (“D-shape”) indicated substantial pressure overload of the right ventricle (RV) in the presence of three high-grade ostial PV stenoses. Distinct systolic notching of pulmonary flow curve was present (**B**). **C**, **D** One day after successful stenting of all three PV stenoses, complete reversibility of LV “D-shape” (**C**) and abolishment of the systolic notch of the pulmonary artery flow curve (**D**) were noted (Additional file 1: video file 1)

A systolic notch being determined on pulmonary arterial flow curve as an indicator of increased pulmonary vascular resistance significantly correlated with depiction of a “D-shape” pattern on cine imaging being indicative of RV pressure overload (p = 0.007; see Fig. 2; Additional file 1). The presence of a systolic notch significantly correlated with the number of stenosed PVs (p = 0.023), while the occurrence of a “D-shape” pattern did not (p = 0.736). In general, both markers of hemodynamic consequences of PV stenosis (“D-shape” pattern and systolic notch) did not correlate with clinical symptoms (NYHA class or hemoptysis).

**Fig. 3:**
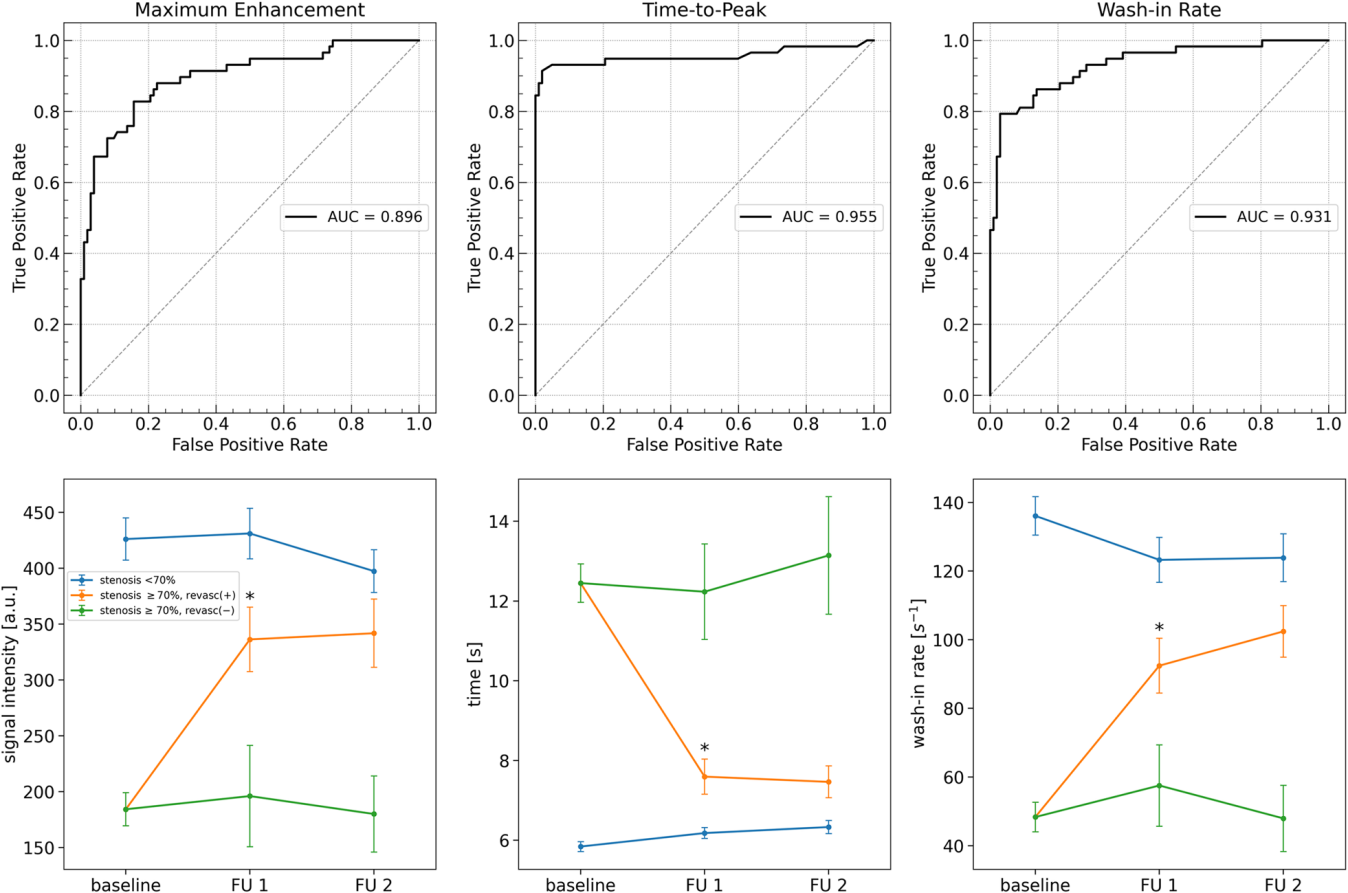
Quantitative analysis of CMR pulmonary perfusion imaging to discriminate ≥ 70% PV stenosis and to determine the gain attributable to successful revascularization. Upper row: ROC analyses were performed to determine the relationship between signal intensity time curve measurements derived from dynamic contrast-enhanced CMR pulmonary perfusion imaging in the presence of ≥ 70% PV luminal diameter stenosis as defined by CMR angiography: time-to-peak enhancement demonstrated the highest discriminatory power resulting in a sensitivity and specificity of 93% and 95%, respectively (cut-off value, 8.0 s). Bottom row: mean change of quantitative measures of pulmonary perfusion at baseline, on day 1 and 3 months after interventional treatment of PV stenosis: for all three quantitative pulmonary perfusion measures, lung lobes with ≥ 70% PV stenosis demonstrated significantly impaired pulmonary perfusion at baseline when compared to non-stenosis dependent lung lobes and successful interventional treatment led to a significant improvement at day 1. In bottom row plots, mean ± standard error of the mean are given. FU 1 indicates follow-up at day 1; FU 2, follow-up at 3 months

Visual assessment of dynamic pulmonary perfusion imaging showed an average perfusion deficit of 1.8 ± 0.9 pulmonary lobes per patient. The lobar perfusion deficits were closely associated with a severe stenosis of the corresponding PV (p < 0.001) with a nearly perfect level of agreement (Cohen’s kappa, 0.96). Quantitative analysis revealed significant differences between lung lobes drained by < 70% vs. ≥ 70% stenosed PVs for maximum enhancement (426 ± 191 vs. 184 ± 113, p < 0.001), time-to-peak enhancement (5.8 ± 1.2s vs. 12.4 ± 3.7s, p < 0.001), wash-in rate (136 ± 57s^−1^ vs. 48 ± 33s^−1^, p < 0.001), and AUC (3629 ± 2001 vs. 1556 ± 1054, p < 0.001), respectively (see Fig. 3). Using the results of CMR angiography, receiver-operating characteristic (ROC) analysis was done to determine the diagnostic value of quantitative pulmonary perfusion measurements for the detection of ≥ 70% PV stenosis: the AUC for time-to-peak enhancement (0.96, 95% CI: 0.91 to 1.0) was best, but wash-in rate (0.93, 95% CI: 0.89 to 0.97) and maximum enhancement (0.90, 95% confidence interval [CI]: 0.84 to 0.95) demonstrated high discriminatory power as well while area-under-the-curve had the lowest relationship (0.84, 95% CI: 0.78 to 0.90); ROC curves are shown in Fig. 3.

**Fig. 4:**
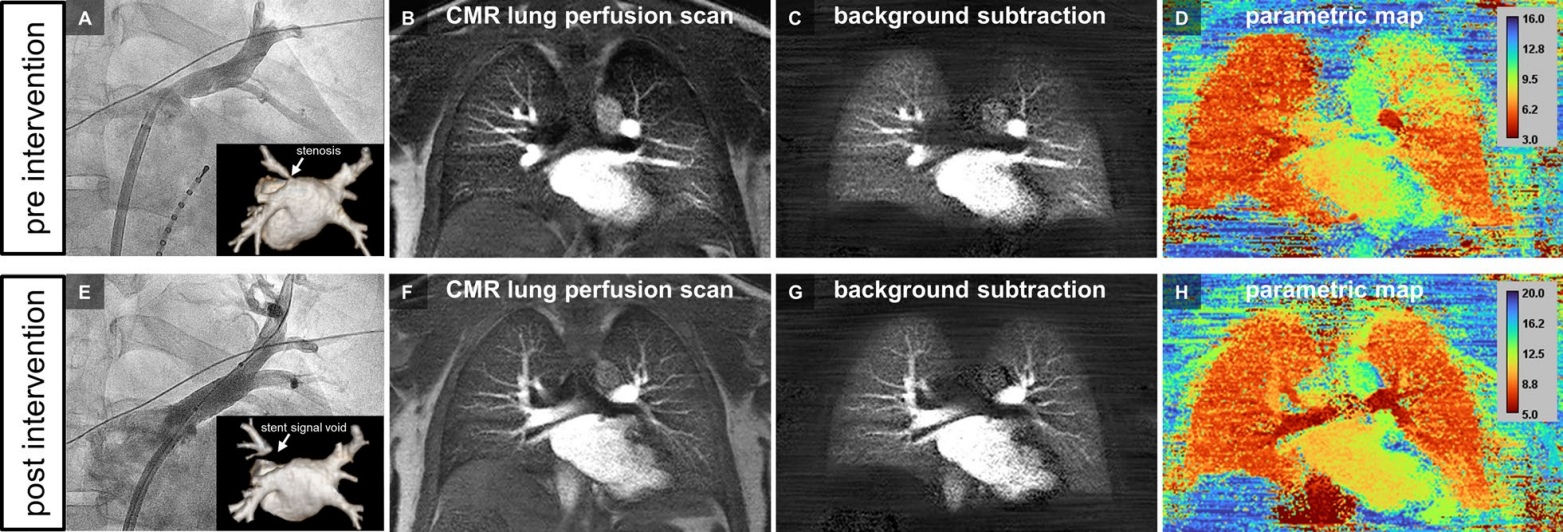
Pre- and post-interventional CMR pulmonary perfusion imaging for assessment of the treatment effect after stenting of a severe ostial left superior pulmonary vein (LSPV) stenosis. Upper row (pre-intervention): Invasive X-ray angiography (**A**, anterior–posterior projection) and pre-interventional CMR angiography (inlay of **A**, posterior–anterior projectional view) revealed a ≥ 70% ostial stenosis of the LSPV (white arrow). CMR pulmonary perfusion imaging depicted a perfusion deficit of the left upper lung lobe (**B**, still frame of original dynamic pulmonary perfusion; **C**, still frame of dynamic pulmonary perfusion after background stationary tissue subtraction; **D**, corresponding pseudo-colored parametric map of quantitative CMR pulmonary perfusion analysis with time-to-peak enhancement as the quantitative measure). Bottom row (post-intervention): Invasive X-ray angiography (**E**, anterior–posterior projection) and post-interventional CMR angiography (inlay of **E**, posterior–anterior projectional view) after successful angioplasty/stenting of PV stenosis with stent-related signal void (white arrow). On post-interventional CMR pulmonary perfusion imaging at day 1, homogenous perfusion of all lung lobes was observed (**F**, still frame of original dynamic pulmonary perfusion; **G**, still frame of dynamic pulmonary perfusion after background stationary tissue subtraction; H, pseudo-colored parametric map of quantitative CMR pulmonary perfusion analysis with time-to-peak enhancement as the quantitative measure; Additional file 2: video file)

**Fig. 5:**
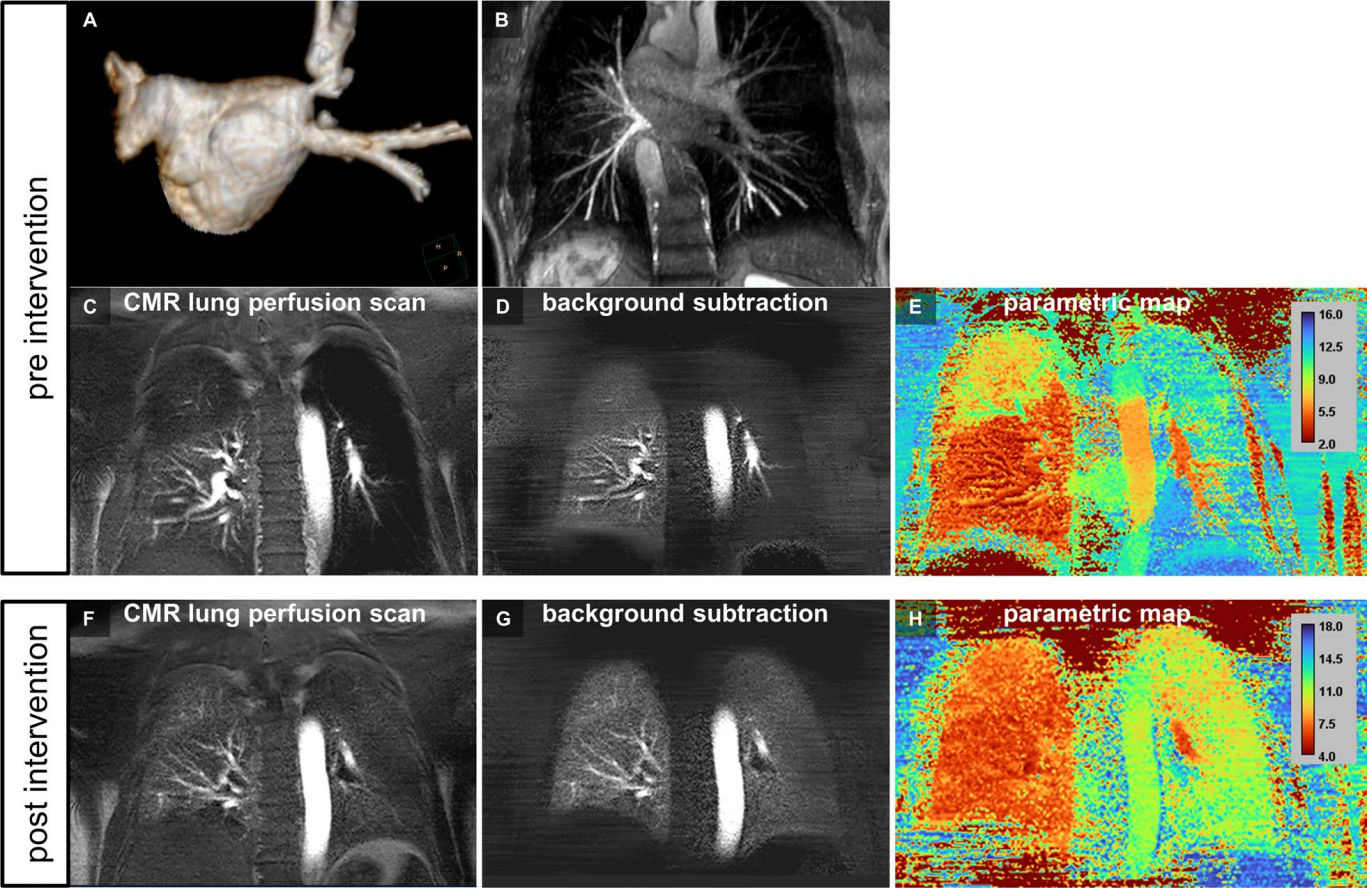
Pre- and post-interventional CMR pulmonary perfusion imaging for assessment of the treatment effect after stenting of multiple severe PV stenoses. **A**, **B** Pre-interventional three-dimensional CMR angiography revealed a total occlusion of both, the LSPV and left inferior pulmonary vein (LIPV; **A**, posterior–anterior view of volume rendering reconstruction) with “late-filling” of large distal pulmonary vein side branches on the second angiographic dynamic scan indicating focal ostial stenosis (**B**, posterior–anterior view of volume intensity projection). In addition, a ≥ 70% luminal diameter stenosis of the right superior pulmonary vein (RSPV) was present. **C–E** Pre-interventional CMR pulmonary perfusion imaging revealed corresponding perfusion loss of the entire left lung and hypoperfusion of the right upper lung lobe (**C**, still frame of original dynamic pulmonary perfusion; **D** still frame of dynamic pulmonary perfusion after background stationary tissue subtraction; **E**, pseudo-colored parametric map of quantitative CMR pulmonary perfusion analysis with time-to-peak enhancement as the quantitative measure). **F**–**H** Post-interventional CMR pulmonary perfusion imaging at day 1 demonstrated improved, but not fully restored perfusion of the left lung while a completely homogenous perfusion of all right lung lobes was seen (**F**, still frame of original dynamic pulmonary perfusion; **G**, still frame of dynamic pulmonary perfusion after background stationary tissue subtraction; **H**, pseudo-colored parametric map of quantitative CMR pulmonary perfusion analysis with time-to-peak enhancement as the quantitative measure; Additional file 3: video file 3)

Figures 4 and 5 provide representative imaging examples of CMR pulmonary perfusion imaging and three-dimensional pulmonary vein CMR angiography (pre vs. post PV revascularization) acquired during the combined single-session protocol.

### Invasive PV angiography and revascularization

Each patient was planned for interventional treatment of at least one PV stenosis depending on stenosis characteristics and time course resulting in a total of 48 targeted PV stenoses. Subsequent invasive X-ray angiography confirmed the results of CMR angiography with high-grade PV stenosis in all 48 PVs (48/48; positive predictive value, 100%). The mean luminal reference diameter of the targeted PVs as measured by CMR angiography was 9.6 ± 2.9 mm. At the discretion of the interventionalist, 35 stenosis were successfully revascularized by stent implantation (35/43, 81%; mean stent diameter, 9.3 ± 1.4 mm), eight stenosis by balloon angioplasty only (8/43; 19%; mean balloon diameter, 6.6 ± 2.3 mm), but in five PV occlusions wire crossing proved to be impossible and revascularization attempt was abandoned (PV reference diameter of successfully vs. unsuccessfully intervened PVs, 9.9 ± 2.8 mm vs. 6.6 ± 2.4 mm; p = 0.014). Overall, acute procedural success rate was 90% (43/48) per targeted pulmonary vein and 94% (30/32) per patient.

### Follow-up evaluation

Patients reported a significant relief of symptoms 3 months after successful PV intervention in comparison to pre-interventional evaluation (NYHA class, 1.6 ± 0.7 vs. 2.8 ± 0.4, p = 0.015). After only one day, the number of patients with a septal D-shape pattern decreased significantly (28% vs. 13%) as well as the occurrence of a systolic notch on pulmonary arterial flow measurement (41% vs. 23%; see Table 1; Fig. 2).

Quantitative pulmonary perfusion analysis revealed large treatment effects one-day post-intervention being attributed to successful PV revascularization as estimated by Cohen’s *d* (time-to-peak enhancement, 1.31 [0.88, 1.74]; maximum enhancement, 0.96 [0.53, 1.39]; wash-in rate, 0.95 [0.52, 1.38]; AUC, 0.93 [0.50, 1.36]; see Figs. 4 and 5, Additional files 2 and 3). Additional gain at 3 months was negligible when compared to one day follow-up as proven by a small treatment effect (Cohen’s *d* for time-to-peak enhancement, 0.07 [− 0.38, 0.52]; maximum enhancement, 0.04 [− 0.41, 0.49]; wash-in rate, 0.22 [− 0.23, 0.67]; AUC, 0.07 [− 0.38, 0.52]). The average gain of time-to-peak enhancement, maximum enhancement and wash-in rate achieved by successful PV revascularization is shown in Fig. 3. Please see Additional file 4 for a side-by-side comparison of pre- and post-intervention color-encoded CMR lung perfusion maps at baseline and 3 months follow up of all 27 patients.

No significant differences between PV stenosis treated by stenting vs. PTA only were seen on quantitative pulmonary perfusion imaging one day post intervention (time-to-peak, 7.4 ± 2.6 vs. 8.4 ± 4.2, p = 0.383; maximum enhancement, 339 ± 179 vs. 325 ± 245, p = 0.853; wash-in rate, 91.0 ± 50.4 vs. 98.6 ± 64.5, p = 0.719; AUC, 2991 ± 1588 vs. 3671 ± 3439, p = 0.397) or 3 months post intervention (time-to-peak, 7.1 ± 2.4 vs. 8.8 ± 2.6, p = 0.102; maximum enhancement, 340 ± 176 vs. 350 ± 255, p = 0.895; wash-in rate, 100.1 ± 43.9 vs. 111.6 ± 60.3, p = 0.543; AUC, 3063 ± 1713 vs. 4340 ± 3300, p = 0.135), respectively.

## Discussion

The main findings of the current CMR study in patients scheduled for interventional treatment of symptomatic PV stenoses were as follows: 1) CMR pulmonary perfusion imaging could be easily integrated into a combined, single-session CMR examination of cine, flow and three-dimensional PV angiography, 2) interventional treatment of PV stenosis aiming at a high proportion of stenting achieved an initial procedural success rate of 90% per targeted vein and 94% per patient, respectively, 3) agreement between visually evaluated pulmonary perfusion imaging and the presence or absence of a severe PV stenosis was nearly perfect, 4) quantitative pulmonary perfusion analysis reliably differentiated lung lobes drained by severely stenosed vs. non-stenosed PVs, with 5) time-to-peak enhancement representing the optimal parameter for the prediction of ≥ 70% PV stenosis, and 6) the average gain attributale to successful PV revascularization as determined by quantitative pulmonary perfusion analysis corresponded to a large treatment effect and occurred already at day 1 post-intervention while additional improvement after 3 months was only marginal.

Radiofrequency catheter ablation is a widespread and increasingly used therapeutic option for patients with atrial fibrillation. The post-procedural development of PV stenosis represents one of the most serious complications with a reported incidence of 1–3% [2, 9]. The spectrum of clinical presentation varies widely, but in general the severity of symptoms depends on the degree of stenosis, the number of affected PVs and the time course of stenosis development [2]. So far diagnosis and pre-procedural stratification is usually based on multiple examinations using echocardiography, CT/CMR angiography and radioisotopic ventilation/perfusion scanning. CT and CMR angiography are commonly considered equivalent with regard to the anatomical assessment of the PVs including side branch depiction and diameter measurements and their usage mainly depends on regional availability and center experience [12, 13]. When compared to invasive X-ray angiography, non-invasive assessment is known to overestimate the degree of PV stenosis, especially in high-grade stenosis and occlusions, respectively [3, 14]. Radioisotopic ventilation/perfusion scans are sensitive, but not specific for PV stenosis, with perfusion abnormalities frequently occurring in lung lobes drained by a severely narrowed PV while corresponding ventilation abnormalities are scarce [15, 16].

CMR imaging offers the unique opportunity to combine imaging modules in order to gather the necessary anatomical, functional and hemodynamic information in one single-session examination without radiation exposure: RV function assessment, pulmonary arterial flow measurement, pulmonary perfusion imaging and three-dimensional PV angiography can be easily integrated into a single CMR protocol resulting in an overall examination duration of less than 30 min.

While CMR represents the acknowledged reference standard for ventricular volume and function assessment and 3D CMR angiography is well established for visualization of PV anatomy prior to catheter ablation procedures, CMR pulmonary perfusion was so far missing within the diagnostic armamentarium of CMR imaging for PV stenosis assessment. Currently, the hemodynamic severity of PV obstruction in children or adults with congenital heart disease is mainly determined by flow measurements in the PVs and arteries in the affected and unaffected lungs [17, 18]. Altered flow profiles, increased peak flow velocities or flow redistribution have been used as indicators of significant PV obstruction [17, 19, 20]. With the addition of CMR pulmonary perfusion imaging depicting the pulmonary microcirculatory level as proposed in the current study, further improvements regarding diagnosis and stratification of congenital heart disease patients can be expected in the future. Previous reports dealt with the applicability of CMR pulmonary perfusion to assess the functional consequences of pulmonary embolism but there is only limited data utilizing CMR pulmonary perfusion imaging for the functional assessment of iatrogenic PV stenosis [5, 21, 22]. In the current study, dynamic CMR pulmonary perfusion was introduced for the pre- and post-interventional evaluation of PV stenosis. In general, pulmonary perfusion imaging relies on the signal enhancement on the parenchymal level resulting from both, pulmonary arterial blood supply and venous drainage. Thus, in patients with PV stenosis or occlusion, contrast-enhanced CMR pulmonary perfusion primarily visualized the hemodynamic consequences of a restricted drainage with decreased or absent parenchymal signal of the corresponding lung lobe. For clinical routine usage, pulmonary perfusion imaging should always be combined with CMR angiography for verification of PV stenosis or PA embolism, respectively, with the target vessel territory chosen according to patient’s history and clinical suspicion.

Visual evaluation of CMR pulmonary perfusion imaging clearly delineated relative hypoperfusion of lung lobes being drained by ≥ 70% PV stenosis as evidenced by a nearly perfect agreement. Quantitative analysis of parenchymal signal intensity time curves resulted in an excellent discrimination of severly stenosed pulmonary veins: time-to-peak enhancement had the highest predictive value for determination of PV stenosis ≥ 70% with a cut-off value of 8.0 s. During follow-up, successful PV revascularization led to a large treatment effect in terms of quantitatively assessed pulmonary perfusion recovery already at day 1 following PV stenting while additional improvement after 3 months was found to be only marginal. It has been previously shown that restoration of normal lung parenchymal hemodynamics and physiology mainly depends on the time to intervention, with the time from diagnosis to revascularization of PV stenosis being a predictor for the post-interventional achievable increase in lung perfusion [23]. In addition, repeated interventions had a positive effect on pulmonary perfusion improvement emphasizing the high incidence of PV re-stenosis [23]. In the current study, the gain in pulmonary perfusion attributable to PV intervention was large already on the first post-procedural day and remained relatively unchanged at 3-months follow-up which can be considered an indicator of the absence of significant early re-stenosis. The high proportion of PV stenting (81%) compared to balloon-angioplasty alone was in accordance with current recommendations and may have contributed to these excellent short-term results. Nevertheless, even with stent implantation, restenosis development may occur in a relevant proportion of patients and should be treated promptly since progression to total occlusion occurs frequently [2]. The proposed combined single-session CMR examination makes it possible to perform radiation-free, highly diagnostic repeat examinations on follow-up visits. In our experience, for routine clinical application a careful visual assessment of pulmonary perfusion based on a side-by-side display of dynamic CMR pulmonary perfusion and colored quantitative perfusion map is usually sufficient for rapid and objective clinical decision-making without the need for detailed analysis of signal intensity time curves during daily routine.

While CMR pulmonary perfusion imaging could verify the direct relationship between severe PV stenosis and decreased perfusion on a parenchymal level, a systolic notch of the pulmonary artery flow curve as an indicator of an increased pulmonary vascular resistance correlated with the number of stenosed PVs. Systolic notching as well as the “D-shape” pattern on cine images resolved after successful revascularization of PV stenosis in a significant number of patients. Disappearance of these hemodynamic markers suggested the transient nature of RV pressure overload and increased pulmonary vascular resistance in patients with PV stenosis after timely performed, successful stenting procedures.

### Limitations

The present study introduced CMR pulmonary perfusion as a valuable addition to the diagnostic armamentarium necessary for detailed pre- and post-interventional evaluation of patients with PV stenosis. Hence, only patients with at least one high-grade PV stenosis targeted for revascularization were included to assess the treatment effect achievable by PV revascularization. The relationship between angiographic degree of PV stenosis, pulmonary perfusion impairment and associated symptoms is rather complex and whether CMR pulmonary perfusion imaging can accurately identify patients with (symptomatic) perfusion abnormalities at lower degrees of PV stenosis shall be addressed in future studies.

Revascularization strategy was pre-procedurally defined by CMR angiography with subsequent invasive angiography being restricted to the targeted PVs only. Consequently, while the presence of high-grade PV stenosis was confirmed in all targeted veins, the overall diagnostic accuracy of CMR PV angiography in comparison to invasive X-ray angiography could not be determined in the current study population.

## Conclusion

Incorporating CMR pulmonary perfusion imaging into a combined single-session CMR examination proved to be a time-efficient and valuable tool for the evaluation of patients with acquired PV stenosis during pre- and post-interventional treatment assessment. Thus, integration of CMR pulmonary perfusion imaging into the clinical work-up of patients with PV stenosis can be effectively used for peri-procedural stratification and at follow-up examinations to determine revascularization success or stenosis progression, respectively.

## Supplementary information

**Additional file 1**: Cine CMR for depiction of left-ventricular “D-shape” pattern. Cine short axis view before (A) and after successful stenting of three high-grade PV stenoses (B). Systolic interventricular septal flattening (left-ventricular “D-shape”) indicated substantial right-ventricular pressure overload in the presence of three severe PV stenoses (identical patient as shown in the still frame of Fig. 2). On day one after successful PV stenting, complete reversibility of left-ventricular “D-shape” (B) was noted 

**Additional file 2**: Pre- and post-interventional CMR pulmonary perfusion imaging for assessment of the treatment effect after stenting of a severe ostial left superior pulmonary vein stenosis. Upper row: Balloon inflation during stenting of a ≥ 70% stenosis of the left superior pulmonary vein (A) resulting in complete angiographic patency (B). Center row: Pre-interventional CMR pulmonary perfusion imaging depicted a corresponding hypoperfusion of the left upper lung lobe (C), while a completely homogenous perfusion of all lung lobes could be documented on CMR pulmonary perfusion imaging one day after successful PV stenting (D; identical patient as shown in the stillframe of Fig. 4). Bottom row: Corresponding color-encoded maps of quantitative pulmonary perfusion analysis displaying the time-to-peak enhancement before (E) and after successful PV stenting (F). Prolonged time-to-peak values of the left upper lung lobe in the presence of a ≥ 70% stenosis of the left superior pulmonary vein could be nicely appreciated (E) but returned to normal after successful interventional treatment (F).

**Additional file 3**: Pre- and post-interventional CMR pulmonary perfusion imaging for assessment of the treatment effect after stenting of multiple severe pulmonary vein stenoses. Upper row: Pre-intervention CMR pulmonary perfusion imaging depicted a complete perfusion loss of the left lung and a hypoperfusion of the right upper lung lobe in a patient with total occlusion of both left-sided PVs and a ≥ 70% stenosis of the right superior PV (identical patient as shown in the still frame of Fig. 5; A, dynamic pulmonary perfusion; B, corresponding subtraction images of pulmonary perfusion; C, color-encoded map of quantitative analysis displaying the time-to-peak enhancement). Bottom row: Post-intervention CMR pulmonary perfusion imaging showed a clearly improved,but not yet returned to normal perfusion of the left lung and a recovered homogenous perfusion of the right upper lung lobe one day after successful stenting of all three PVs (D, dynamic pulmonary perfusion; E, corresponding subtraction images of pulmonary perfusion; F, color-encoded map of quantitative analysis displaying the time-to-peak enhancement).

**Additional file 4**: Pre- versus post-intervention color-encoded CMR lung perfusion maps. Side-by-side comparison of pre- and post-intervention color-encoded CMR lung perfusion maps at 3 months follow up (pseudo-colored parametric maps of quantitative CMR pulmonary perfusion analysis with time-to-peak enhancement as the quantitative measure, calibration bar values inseconds, n=27 patients).

## Data Availability

Not applicable.

## Abbreviations

AUC: Area under the curve
BMI: Body mass index
CI: Confidence interval
CMR: Cardiovascular magnetic resonance
CT: Computed tomography
ECG: Electrocardiogram
LIPV: Left inferior pulmonary vein
LSPV: Left superior pulmonary vein
LV: Left ventricle/left ventricular
LVEDV: Left ventricular end-diastolic volume
LVEF: Left ventricular ejection fraction
LVESV: Left ventricular end-systolic volume
NYHA: New York Heart Association
PA: Pulmonary artery
PV: Pulmonary vein
RIPV: Right inferior pulmonary vein
RMPV: Right middle pulmonary vein
ROC: Receiver-operating characteristic
RSPV: Right superior pulmonary vein
RV: Right ventricle/right ventricular
RVEDV: Right ventricular end-diastolic volume
RVEF: Right ventricular ejection fraction
RVESV: Right ventricular end-systolic volume
