# Supplementary material for: Cardiovascular magnetic resonance pulmonary perfusion for guidance of interventional treatment of pulmonary vein stenosis
Source: J Cardiovasc Magn Reson. 2022 Dec 12;24:70. doi: 10.1186/s12968-022-00904-x (PMC9743617; doi:10.1186/s12968-022-00904-x)
Supplement: Supplementary file 4 — Additional file 4: Pre- versus post-intervention color-encoded CMR lung perfusion maps. Side-by-side comparison of pre- and post-intervention color-encoded CMR lung perfusion maps at 3 months follow up (pseudo-colored parametric maps of quantitative CMR pulmonary perfusion analysis with time-to-peak enhancement as the quantitative measure, calibration bar values inseconds, n=27 patients). [file 12968_2022_904_MOESM4_ESM.pdf]

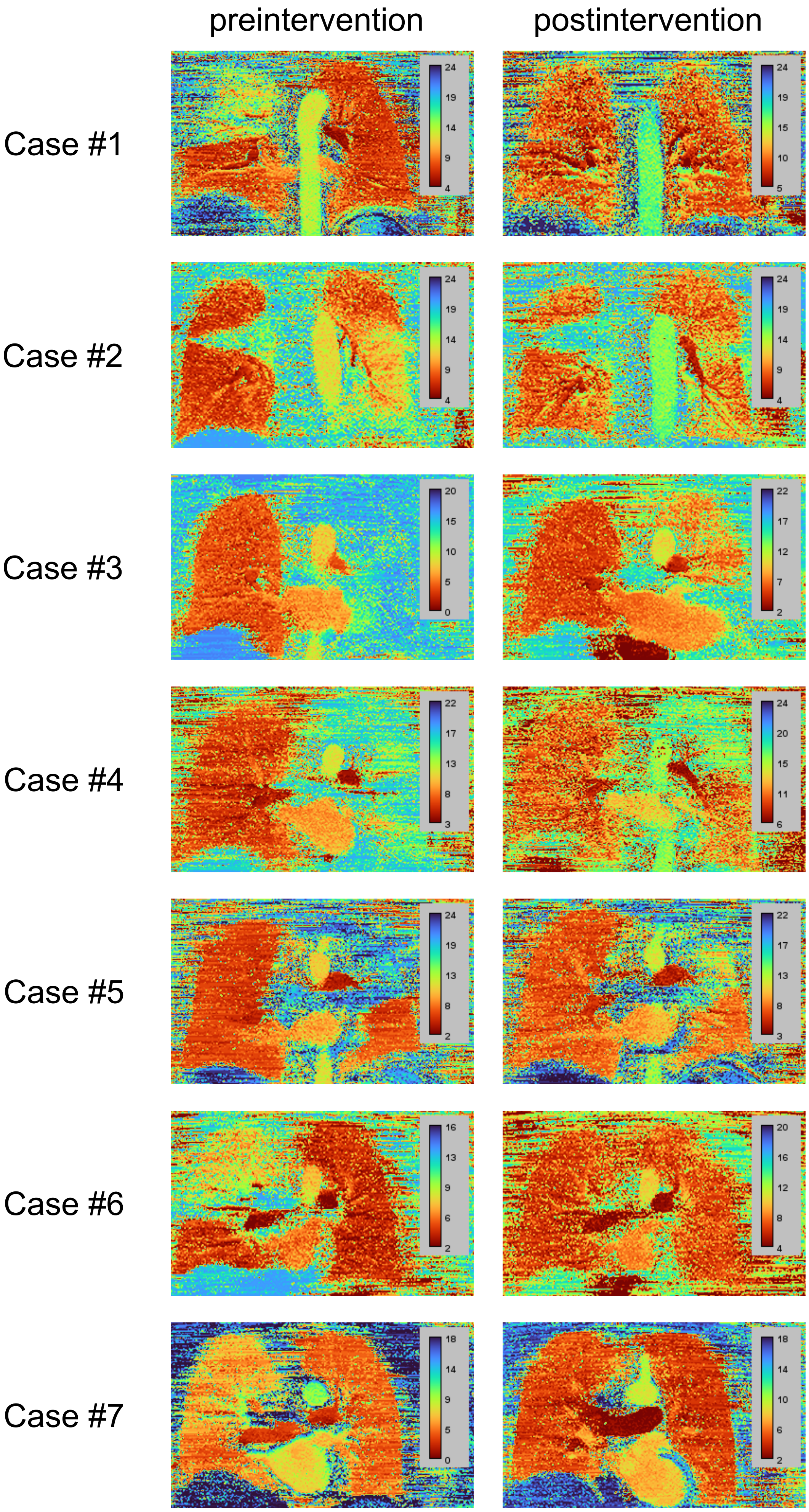
preintervention
postintervention
Case #1
24 19 14 9 4
24 19 15 10 5
Case #2
24 19 14 9 4
24 19 14 9 4
Case #3
20 15 10 5 0
22 17 12 7 2
Case #4
22 17 13 8 3
24 20 15 11 6
Case #5
24 19 13 8 2
22 17 13 8 3
Case #6
16 13 9 6 2
20 16 12 8 4
Case #7
18 14 9 5 0
18 14 10 6 2

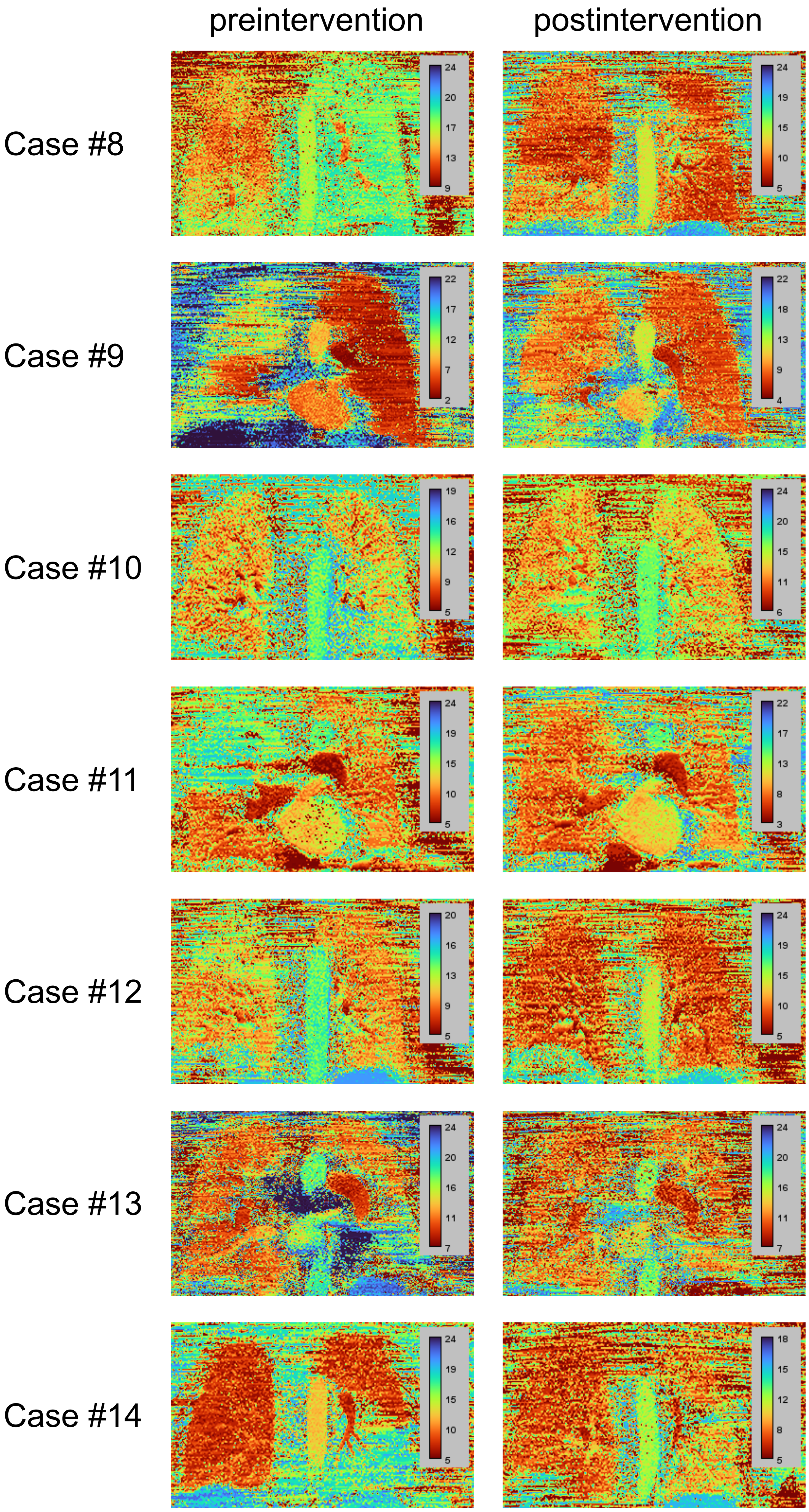
preintervention
postintervention
Case #8
24 20 17 13 9
24 19 15 10 5
Case #9
22 17 12 7 2
22 18 13 9 4
Case #10
19 16 12 9 5
24 20 15 11 6
Case #11
24 19 15 10 5
22 17 13 8 3
Case #12
20 16 13 9 5
24 19 15 10 5
Case #13
24 20 16 11 7
24 20 16 11 7
Case #14
24 19 15 10 5
18 15 12 8 5

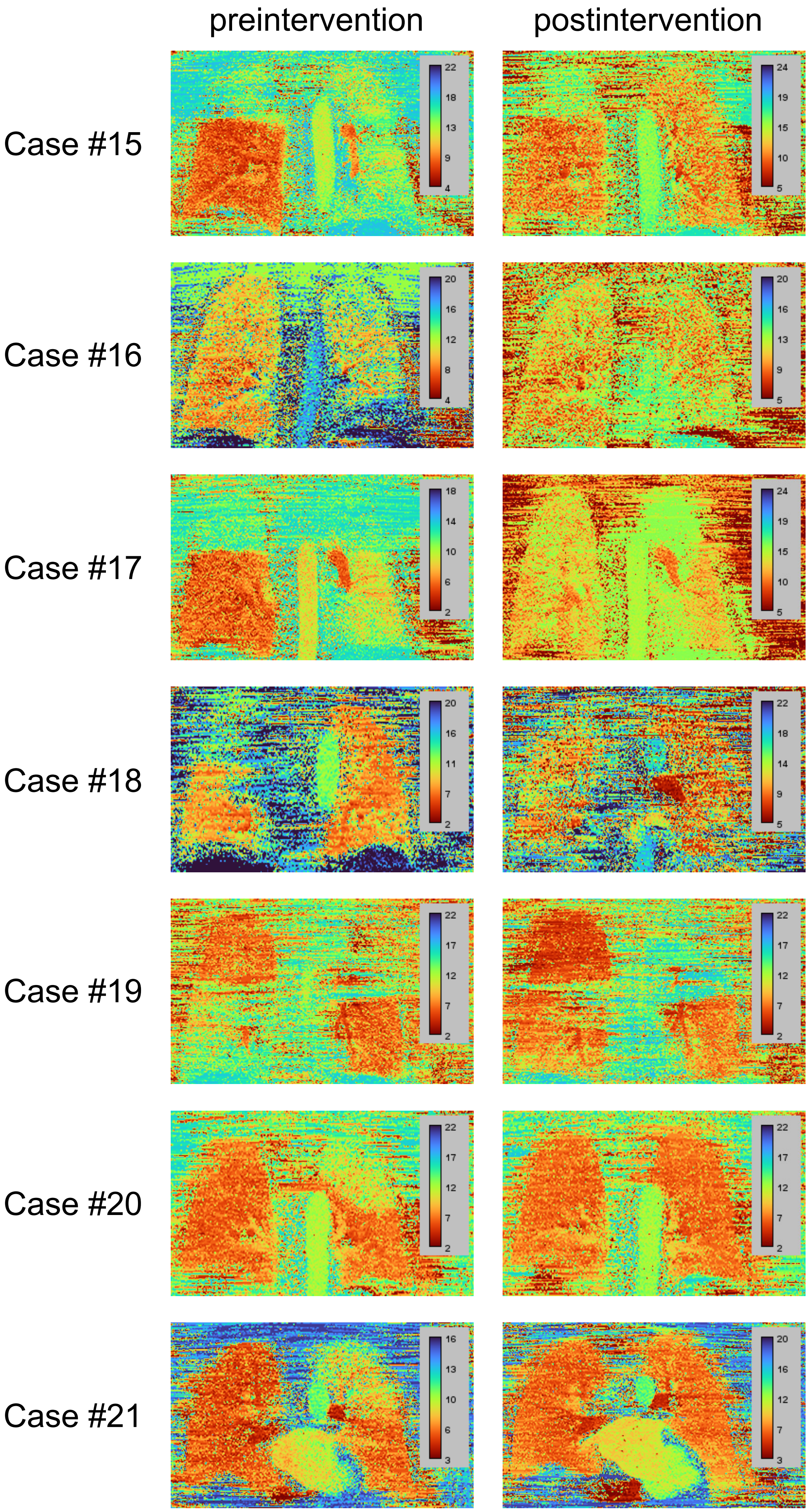
preintervention
postintervention
Case #15
22
18
13
9
4
24
19
15
10
5
Case #16
20
16
12
8
4
20
16
13
9
5
Case #17
18
14
10
6
2
24
19
15
10
5
Case #18
20
16
11
7
2
22
18
14
9
5
Case #19
22
17
12
7
2
22
17
12
7
2
Case #20
22
17
12
7
2
22
17
12
7
2
Case #21
16
13
10
6
3
20
16
12
7
3

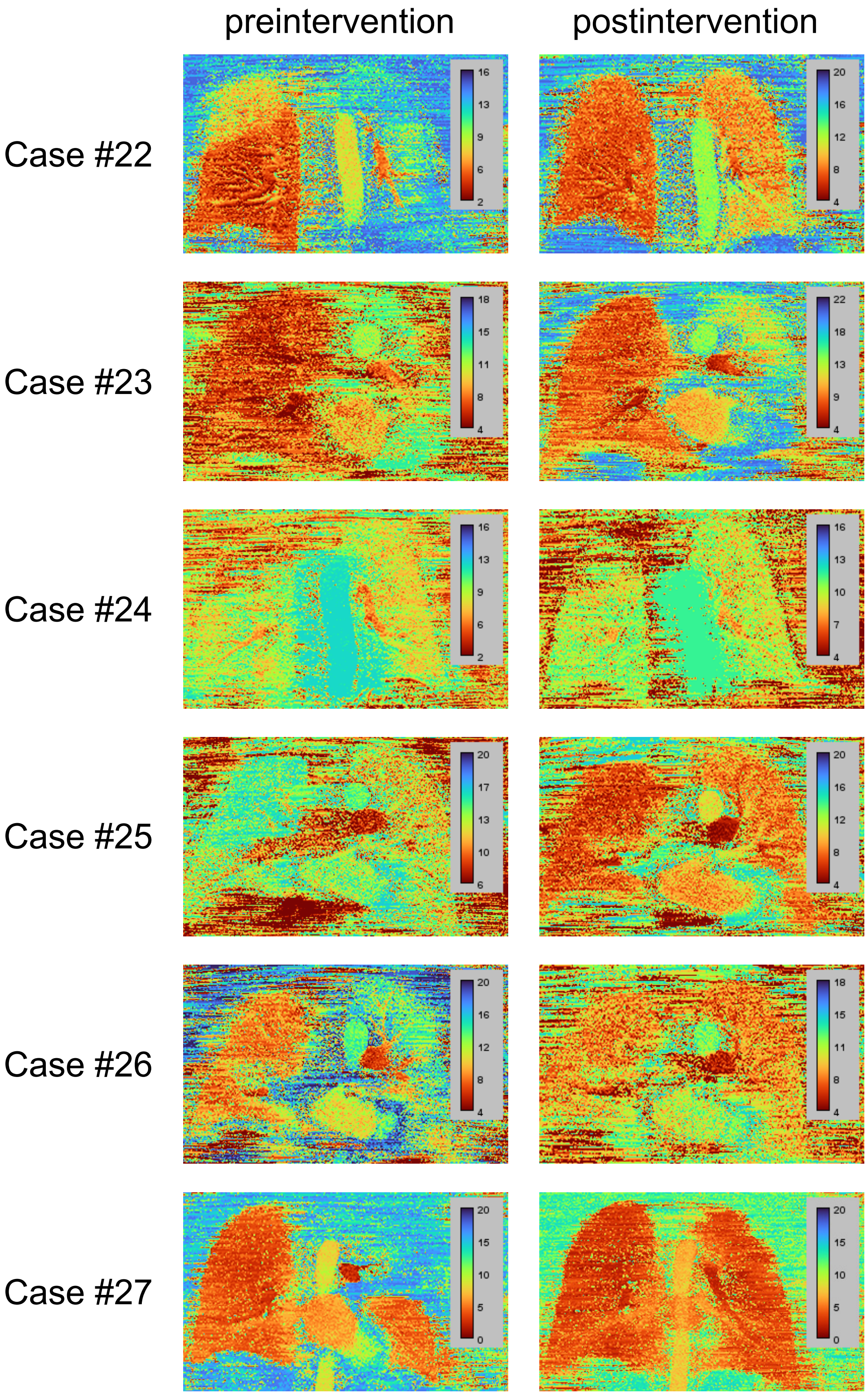
preintervention
postintervention
Case #22
16 13 9 6 2
20 16 12 8 4
Case #23
18 15 11 8 4
22 18 13 9 4
Case #24
16 13 9 6 2
16 13 10 7 4
Case #25
20 17 13 10 6
20 16 12 8 4
Case #26
20 16 12 8 4
18 15 11 8 4
Case #27
20 15 10 5 0
20 15 10 5 0
